# Comparison of liver MRI R2(FerriScan®) VS liver MRI T2* as a measure of body iron load in a cohort of beta thalassaemia major patients

**DOI:** 10.1186/s13023-020-1301-4

**Published:** 2020-01-22

**Authors:** Padmapani Padeniya, Shirom Siriwardana, Dileepa Ediriweera, Nayana Samarasinghe, Sasanka Silva, Ishari Silva, Nizri Ahamed, Madunil Niriella, Anuja Premawardhena

**Affiliations:** 1 Faculty of Medicine, University of Kelaniya, P.O Box 6, Thalagolla Road, Ragama, Sri Lanka; 2 Adolescent and Adult Thalassaemia Care Center (University Medical Unit), North Colombo Teaching Hospital, No. 10, Sirima Bandaranayake Mawatha, Kadawatha, Sri Lanka; 3 Hemas hospital, Wattala, Sri Lanka; 4 Lady Ridgeway Hospital for Children, Borella, Sri Lanka

**Keywords:** Iron overload, FerriScan®, T2* scan, Liver iron concentration, MRI

## Abstract

To compare the similarity of the non-patented T2* and the high cost patented R2 (Ferriscan®) MRI techniques in the measurement of liver iron concentration (LIC) in heavily transfused patients with thalassaemia major in a real- life Sri Lankan hospital setup. We compared LIC measured by MRI, obtained 2 weeks apart, using both T2* and R2 techniques in 15 patients with beta thalassaemia major. They all had a history of > 100 units of blood transfusions life long and also a history of sub optimal chelation. MRI R2 and MRI T2* scan values showed a negative correlation (co-rrelation coefficient = − 0.63, *p* = 0.01) This correlation was strong in lower LICs and progressively decreased with upper LIC values. Thus a significant discrepancy was observed between median values of two MRI technologies (*p* = 0.0005) with T2* tending to underestimate iron overload especially in those with very high LIC identified by R2. The lack of concordance of T2* and R2 especially in those with very high reading on R2 suggest the potential errors in interpretations that can occur in “non-expert centres”; which are likely to lead to errors in clinical judgement on the intensity of chelation therapy needed.

## Introduction

Complications due to iron overload continues to be a leading cause of morbidity and mortality in patients with transfusion dependent thalassaemia [1, 2]. This is no exception in the 2000 or so patients with thalassaemia who attend transfusion centres in Sri Lanka [3]. Liver is a major organ of iron deposition ensuing liver parenchymal damage, fibrosis, cirrhosis, liver failure and hepatocellular carcinoma [3].

Serum ferritin is the most widely used method for assessing body iron overload in patients with transfusion dependent thalassaemia. It is relatively inexpensive therefore could be used repeatedly. However, serum ferritin is unreliable in many clinical situations [4, 5]. In transfusion dependent anaemia, liver iron concentration (LIC) reflects total body iron content and serves as an indirect measurement of total body iron burden. Therefore, liver biopsy remains the reference method of assessing LIC to date. However, its invasive nature and owing to significant sampling variability researchers were seeking for non-invasive alternatives [2, 4]. .Amongst non-invasive methods, MRI based methods are robust, fast, can be used repeatedly and have been shown to be accurate over the entire clinically relevant ranges of LIC [2]. Two MRI methods concern in this study were based on two different data acquisition methods. FerriScan® / MRI R2 is a FDA approved commercial product which uses the R2 technique to estimate LIC. This has been validated against liver biopsies. However, wider use is limited due to the high cost [5, 6]. MRI T2* based methods are available for free and are widely used due to the free availability of the software. Used by specialists in the field T2* has being shown to be reliable [7]. The free access to the software has allowed T2* technique to be widely used in non-specialist centers with no sound back ground with calibration and usage of the tool.

The objective of this study was to compare LIC values obtained from MRI R2 (FerriScan®) and MRI T2* techniques in the same group of heavily transfused beta thalassaemia major patients and find the association of the LIC values obtained by these two applications. Further the study compares the serum ferritin values of the same individuals with their LIC counterparts obtained by MRI R2 and MRI T2* applications. This study is a part of a major study which was originally designed to estimate the reversibility of liver fibrosis in patients with beta thalassaemia major with aggressive chelation therapy.

## Methods

A prospective cross sectional study was carried out in patients who are registered at the Adult and Adolescent thalassaemia unit Kiribathgoda, Sri Lanka. Ethical clearance was obtained from the Ethics Review Committee (ERC) of the Faculty of Medicine, University of Kelaniya, Sri Lanka and the study was performed in accordance with the declaration of Helsinki.

Beta thalassaemia major patients who have undergone more than 100 blood transfusions life time with elevated serum ferritin > 2000 ng/mL on three consecutive values done 3 months apart were selected for the study. The liver MRI R2 scan was available at a private sector hospital (Hemas Hospital Wattala, Sri Lanka) which had set up in collaboration with the Resonance Health Limited, Australia. The protocol was followed up in accordance with the manufacturer’s instructions [6]. The MRI scanner which was set up for the R2 analysis was a Philips multiva 1.5 T machine with an echo time of 1 ms. The slice thickness was 6 mm, the matrix size was 256 mm and the fat suppression was not used. The liver MRI T2* was already established in the Lady Ridgeway Hospital for Children, Borella, Sri Lanka. This centre had already over two-year experience in using the technique. This scanner is a Siemens 1.5 T machine with an echo time of 1.3 ms and a repetition time of 200 ms. The slice thickness used in the MRI T2* was 10 mm, the matrix size was 2.7*10 mm and fat suppression was not used. The LIC calculations were based on Hankins JS et al. 2009 and MRI T2* was converted to reciprocal R2* by the formula; R2*[Hz] = 1000/T2*[ms] [8].

At the time of recruitment blood was collected from all the study participants to assess the serum ferritin level. Serum ferritin was measured by a solid-phase, two site chemiluminescent enzyme immunometric assay in the North Colombo (Teaching) Hospital. All the study participants were then subjected to liver MRI R2 and liver MRI T2* scans within 2 weeks’ interval to assess the liver iron concentration from two different non-invasive approaches.

The final ferritin value at the time of recruitment was considered for the analysis. Distribution of the continuous variables like age, serum ferritin and LIC, were presented with mean and the standard deviation. The Mann Whitney U test was used to compare the medians between two test values as the data were not normally distributed. The *p* value < 0.05 was considered to be statistically significant. Data were analyzed with R programming language version 3.4.2.

## Results

Total of 15 beta thalassaemia major patients were recruited for the study. 10 (66.6%) were males. The mean age of the study population was 21 years (Standard deviation/SD = 4.3) (range 15–28 years). The mean serum ferritin level was 3126 ng/mL (SD = 911) (range 1540–4540). Except three patients all the others had serum ferritin levels greater than 2500 ng/mL.(Additional file 1 Table S1 is annexed).

**Fig. 1:**
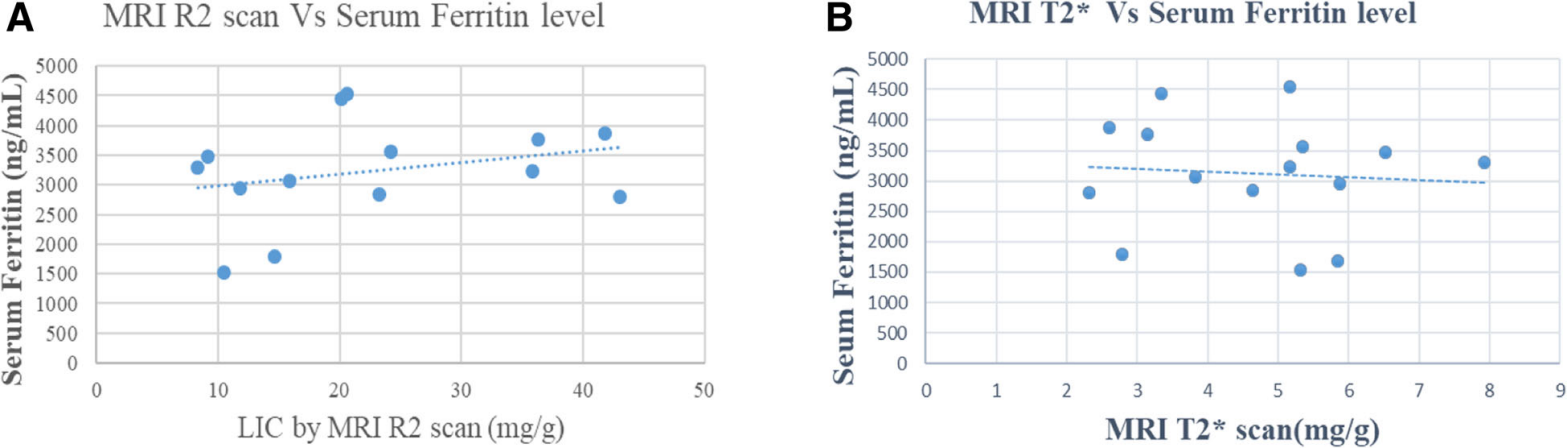
**a**: Correlation between MRI R2 and Serum ferritin. **b**: Correlation between MRI T2* and Serum ferritin

Serum ferritin and MRI R2 scan values did not show a linear correlation (correlation coefficient = 0.368, *p* = 0.177) (Fig. 1a); Nor was there a liner correlation between serum ferritin and MRI T2* scan values (correlation coefficient = 0.114, *p* = 0. 685) (Fig. 1b).

**Fig. 2:**
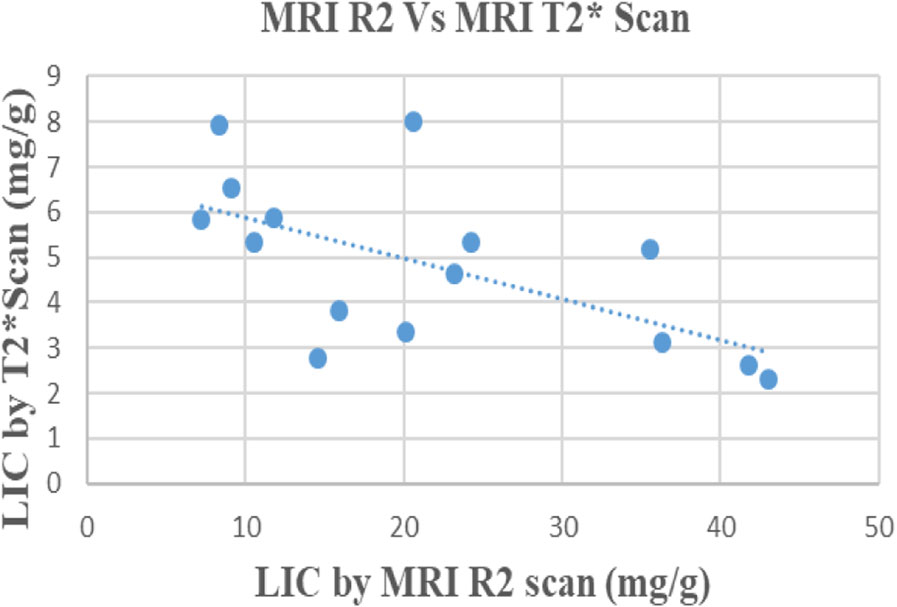
Correlation between MRI R2 and MRI T2* scan

MRI R2 and MRI T2* scan values showed a negative correlation (correlation coefficient = − 0.63, *p* = 0.01) (Fig. 2). This correlation was strong in lower LICs and progressively decreased with upper LIC values.

**Fig. 3:**
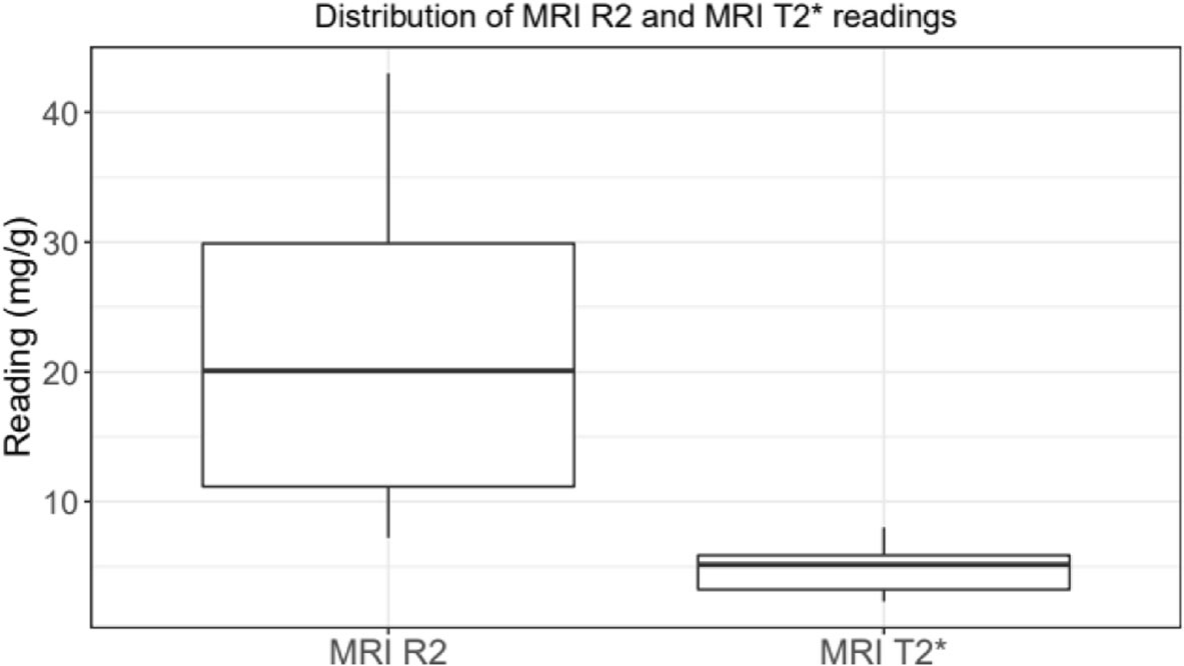
Box plot of MRI R2 and MRI T2* values distribution (mg/g)

Median LIC obtained by the MRI R2 and MRI T2* are 20.10 mg ((Q_1_ = 11.15; Q_3_ = 29.90) Fe/g dry tissue and 5.16 mg (Q_1_ = 3.24; Q_3_ = 5.85) Fe/g dry tissue respectively. Median value obtained by MRI T2* scan was significantly lower than the median value achieved by the MRI R2 scan (*p* = 0.0005) (Fig. 3).

## Discussion

Though liver biopsy is the gold standard method of assessing LIC FerriScan® is being used as an alternative for liver biopsy and LIC measurement as it has been proven to be accurate and reproducible in multiple settings. In this comparative study of LIC values using two different MRI protocols of the same set of patients we observed a very wide discrepancy of the values. The R2 scan was able to identify a wide range of LICs (7-43 mg/g) whilst with T2* the scatter was quite limited (all had < 8 mg/g) (Fig. 2). There was an inability of T2* to capture alarmingly high iron overloads identified by R2 scan.

The deficiency of T2* technique may merely suggest the lack of expertise of the center to use the technology rather than an inherent defect of the technology itself as T2* in specialized centers has shown reliable LIC measurements [9, 10]. Our study suggests that in the present setting in Sri Lanka, the cheaper technique available at present to measure body iron namely serum ferritin and MRI T2* is proving to be unreliable specially in those who are at the biggest danger from iron toxicity; namely the heavily transfused highly iron loaded patients. A study recruiting over a 1000 patients in Vietnam comparing T2* and R2 has demonstrated similar findings to this study which necessitated for the Thalassaemia International Federation to release a clinical alert [11].

We reiterate the need for more research for the development of cheaper non -invasive techniques to measure body iron load which could be widely used safely and reliably in patients with thalassaemia. Until such techniques are available the clinicians using the currently available techniques should be aware of the limitations of the current technologies to avoid erroneous judgments.

This study has several limitations. To compare any two techniques, it’s imperative that both are compared against the gold standard. In this case this would have entailed doing liver biopsies and measuring LIC. This was a not part of our study design and we had no consent nor the technical expertise for LIC measurement from liver tissue. Another limitation in the study was the two-week time period between MRI R2 scan and the MRI T2* scan. Simultaneous measurements would have made the comparison ideal. As the machines were located separately under two different administrative institutions the practical issues prevented us from doing this. The small sample size is also acknowledged by us as a limitation.

Despite all the limitations this study highlights the errors in clinical judgement that can befall if a clinician is not aware about the limitations of the technologies that are at his disposal.

## Supplementary information

**Additional file 1: Table S1.** Distribution of serum ferritin, MRI R2 and MRI T2* values in the study group.

## Data Availability

All data generated or analyzed during this study are included in this published article [and its supplementary information files].

## Abbreviations

ERC: Ethics review committee
LIC: Liver iron concentration
MRI: Magnetic resonance image
SD: Standard deviation
