# Supplementary material for: Comparison of liver MRI R2(FerriScan®) VS liver MRI T2* as a measure of body iron load in a cohort of beta thalassaemia major patients
Source: Orphanet J Rare Dis. 2020 Jan 22;15:26. doi: 10.1186/s13023-020-1301-4 (PMC6977251; doi:10.1186/s13023-020-1301-4)
Supplement: Supplementary file 1 — Additional file 1: Table S1. Distribution of serum ferritin, MRI R2 and MRI T2* values in the study group. [file 13023_2020_1301_MOESM1_ESM.pdf]

Patient no	*Ferritin* Levels (ng/mL)	MRI R2 value mg/g (mmol/kg)	MRI T2 * value (mg/g)
1. RTC 57	2800	>43.0mg/g (>770)	2.32mg/g
2. RTC 317	3870	41.8 mg/g (749)	2.61mg/g
3. RTC 07	3770	36.3 mg/g (650)	3.14mg/g
4. RTC 327	3240	35.6mg/g(637)	5.16mg/g
5. RTC 357	3560	24.2 mg/g (433)	5.34 mg/g
6. RTC 100	2850	23.2 mg/g (416)	4.64 mg/g
7. RTC 120	4540	20.6mg/g(369)	5.16mg/g
8. RTC 281	4440	20.1mg/g (361)	3.34 mg/g
9. RTC 256	3070	15.9 mg/g (286)	3.83 mg/g
10. RTC 311	1800	14.6mg/g (261)	2.78mg/g
11. RTC 318	2950	11.8 mg/g (211)	5.87 mg/g
12. RTC 340	1530	10.5 mg/g (187)	5.32 mg/g
13. RTC 321	3480	9.1 mg/g (164)	6.52mg/g
14. RTC 229	3300	8.3 mg/g (148)	7.93 mg/g
15. RTC 367	1680	7.2 mg/g (129)	5.84mg/g

Supplementary Table 1: Distribution of serum ferritin, MRI R2 and MRI T2* values in the study group.
